# A cross-sectional self-assessment of burnout amongst a sample of doctors in Ghana

**DOI:** 10.4102/phcfm.v12i1.2336

**Published:** 2020-08-19

**Authors:** Nana K. Ayisi-Boateng, Elizabeth M. Bankah, Gerhard K. Ofori-Amankwah, Dora A. Egblewogbe, Emmanuel Ati, Douglas A. Opoku, Emmanuel Appiah-Brempong, Kathryn Spangenberg

**Affiliations:** 1 Department of Medicine, School of Medicine and Dentistry, Kwame Nkrumah University of Science and Technology, Kumasi, Ghana; 2 Department of Family Medicine, Greater Accra Regional Hospital, Accra, Ghana; 3 Department of Medical Services, FOCOS Orthopaedic Hospital, Accra, Ghana; 4 Polyclinic/Family Medicine Sub BMC, Korle Bu Teaching Hospital, Accra, Ghana; 5 Family Medicine Directorate, Komfo Anokye Teaching Hospital, Kumasi, Ghana; 6 School of Public Health, Kwame Nkrumah University of Science and Technology, Kumasi, Ghana

**Keywords:** burnout, depersonalisation, exhaustion, Ghana, physician

## Abstract

**Background:**

The occurrence of burnout amongst African health professionals has been widely anticipated, but there is a dearth of published data, especially amongst doctors. Burnout has been reported to be as high as 53% amongst doctors in the United States. If not detected, it can result in prescription errors, work-related accidents, substance abuse and depression.

**Aim:**

The aim of this study was to determine the prevalence of burnout and its associated factors amongst a sample of physicians in Ghana.

**Setting:**

This study was conducted in Kumasi amongst physicians attending a conference organised by the West African College of Physicians, Ghana Chapter.

**Method:**

A cross-sectional study. Of the 90 physicians who registered for the conference, 60 responded to a self-administered Maslach Burnout Inventory questionnaire. Data were analysed descriptively and inferentially using STATA^®^ version 14.

**Results:**

Approximately 52% of respondents had been in medical practice for 10–19 years (mean 15.4 years). All the major medical specialties were represented. Internal Medicine had the highest number of participants (48.3%). With respect to the components of burnout, 5.5% of respondents experienced depersonalisation, 7.8% had a lack of personal achievement and 10.8% experienced emotional exhaustion. The association between burnout and age, sex, years of practice and clinical specialty was not found to be statistically significant.

**Conclusion:**

This pilot study has shown burnout to be common amongst physicians in Ghana. It is recommended that further studies are conducted, involving a larger cross-section of doctors in various parts of Africa.

## Introduction

Burnout, as defined by Maslach, is a syndrome that involves overwhelming mental and psychological exhaustion, a decreased sense of accomplishment and self-esteem and depersonalisation (detachment from the job).^1^ Burnout is a common phenomenon amongst those whose occupation involves constant contact with people.^2^ Although working with people may be gratifying, the stress of dealing with peoples’ heightened emotions on a regular basis has the potential for psychological burden^1^ which, if not handled appropriately, may lead to burnout. Burnout is multifactorial and often signifies the inability of an individual to cope with work demands and interpersonal stressors at work.

Emotional exhaustion may manifest as fatigue, loss of energy, a feeling of helplessness or loss of enthusiasm. Depersonalisation may be noticed when a physician develops negative work attitude and begins to treat patients like objects. Similarly, lack of accomplishment may manifest as difficulty coping with work and decreased work productivity.^3,4^ Physicians who experience burnout are at risk of suicidal ideation, developing alcohol use disorders and dysfunctional interpersonal and work-related relationships. Adverse effects on quality of care and the risk for medical errors are other important complications that may result from burnout.^5^

Worldwide, the incidence of physician burnout is increasing and studies have estimated a lifetime risk of burnout in one-third of practicing physicians.^6^ In a study on burnout and satisfaction with work–life balance amongst US physicians relative to the general US population, 45.8% of physicians reported at least one symptom of burnout.^6^ Physicians in Ghana, and Africa in general, are faced with a myriad of challenges. Overcrowded outpatient departments, emergency rooms, specialist clinics and wards with inadequate doctor-to-patient ratios are common.^7,8^ Hospitals and clinics generally lack essential diagnostic equipment, medications and supporting staff.^7,9^ In addition, low remuneration, poor work–life balance, challenges with postgraduate training and identification of mentors to provide guidance prevail.^10^ These potentially put stress on doctors regularly. Although these factors predispose physicians to burnout, it is difficult to identify a specific cause.

In a South African study, 81% of physicians had clinically significant burnout.^11^ In Jos Plateau, Nigeria, a study amongst primary care physicians showed that 34.8% had a high degree of burnout.^12^ Patel et al. in a review cited work-related factors contributing to burnout as excessive workloads, long working hours, frequent night and weekend calls, and time spent at home.^13^ Even though some of these factors are anticipated to prevail amongst doctors in Ghana, the literature on it is lacking.

We, therefore, conducted this pilot study to assess the prevalence of burnout amongst a sample of physicians in Ghana. It was also to determine whether there is any association between burnout and factors such as age, gender, medical specialty and years of practice.

## Research methods and design

### Study design and setting

This was a cross-sectional study carried out at the Annual General Scientific Meeting (AGSM) of the West African College of Physicians (WACP), Ghana Chapter, held in Kumasi, Ghana, on 26 July 2019.

### Study population

Participants were physicians drawn from the following disciplines: community health, family medicine, internal medicine, laboratory medicine, psychiatry and paediatrics. A member of the WACP, also known as a specialist, has sat for and passed a postgraduate certificate examination in a chosen specialty area, whilst a fellow, or senior specialist or consultant, has either gone through a certification examination or been elected to the rank. The WACP, Ghana Chapter, has approximately 150 members and fellows. Out of this, 90 physicians registered and attended the AGSM.

### Sampling

To reduce the probability of type 2 error to the barest minimum and to ensure at least 80% statistical power, a sample size of 136 was estimated. However, 90 physicians registered for the WACP-Ghana conference. Accidental sampling technique was used in which only eligible participants present at the conference venue and consented to participate in the study were recruited. Although accidental sampling limits the external validity (i.e. generalisability) of study results, the technique is acceptable in a pilot study like ours where an enquiry is preliminary and intended to pave a way for a more structured and robust study.

All the 90 conference participants were informed about the study and invited to participate by filling out a questionnaire. Copies of the questionnaire were put in participants’ conference files which were handed over to each of them as they entered the conference venue.

### Data collection

All data were collected using a questionnaire. The questionnaire had a section for socio-demographic data of participants and also included the Maslach Burnout Inventory – Human self-test (MBI-HST).^1^ Participants were invited to respond to the questionnaire after one of the authors had presented on the subject of burnout and explained the MBI-HST tool and its interpretation. The inventory assesses burnout in three main domains, including exhaustion, depersonalisation and personal achievement. Sub-items were used for measuring the domains as follows: ‘exhaustion’ domain – seven items, ‘depersonalization’ domain – seven items and ‘personal achievement’ domain – eight items. Each item was scored on a 7-point Likert scale ranging from 0 to 6, with higher values representing higher frequencies for an item. The descriptive terms of the scale are ‘never’ – 0, ‘a few times per year’ – 1, ‘once a month’ – 2, ‘a few times per month’ – 3, ‘once a week’ – 4, ‘a few times per week’ – 5 and ‘every day’ – 6.

The cumulated scores for each domain were computed for each respondent. The ‘exhaustion’ domain was classified as severe burnout for scores ≥ 30, moderate burnout for scores ranging 18–29 and low-level burnout for scores ≤ 17. For the ‘depersonalization’ domain, scores ≥ 12 were described as high-level burnout, scores from 6 to 11 as moderate burnout and scores < 6 as low-level burnout. The scores for ‘personal achievement’ domain were described as follows: ≥ 40 as low-level burnout; 34–39 as moderate burnout and ≤ 33 as high burnout. Socio-demographic data collected included years of service, gender and sub-specialties. At the end of the 1-day conference, a research assistant collected completed questionnaires from participants. Data were initially collected and cleaned in Microsoft Excel and then imported into Stata 14 software for analysis. Analysis included univariate and bivariate analyses. Univariate analysis was performed for each socio-demographic data and each domain of burnout. Bivariate analysis with linear regression models was done to assess the relationship between continuous socio-demographic variables and the various domains of burnout. Chi-square test and regression analysis of the independent variables was also performed to assess the relationship between categorical socio-demographic variables and various domains of burnout.

### Ethical consideration

The study was conducted as part of conference proceedings. Verbal consent was obtained from participants whilst the conference was in session and the questionnaires distributed. Confidentiality and anonymity were ensured as names of participants were not required on the questionnaires and other study-related documents. Approval for the study was obtained from the West African College of Physicians (WACP).

## Results

### Characteristics of study participants

Out of 90 physicians who attended the conference, 60 returned the completed questionnaires, representing a response rate of 66.67%. Most participants were between ages 35 and 44 years (43.3%). The minimum number of years a physician had practised was 3 and the maximum was 43, with a mean of 15.4 years (standard deviation [s.d.] ± 8.7). The specialty with the highest number of respondents was internal medicine (48.3) and the lowest were laboratory medicine and psychiatry (Table 1).

**TABLE 1:** Socio-demographic characteristics of respondents.

Variable	Frequency (*n*)	%
**Age (years)**		
25–34	13	21.7
35–44	26	43.3
45–54	14	23.3
55–64	5	8.3
> 64	2	3.3
Mean age (years) [s.d.]	15.4 [±8.7]	-
**Sex**		
Male	30	50.0
Female	30	50.0
**Number of years of practice**		
1–9	17	28.3
10–19	31	51.7
20–29	7	11.7
30–39	3	5.0
40–49	2	3.3
**Specialty**		
Community health	3	5.0
Family medicine	8	13.3
Internal medicine	29	48.3
Laboratory medicine	2	3.3
Paediatrics	16	26.3
Psychiatry	2	3.3

s.d., standard deviation.

### Burnout components

Majority of the respondents reported low-level burnout across all three domains. Amongst the respondents, 8.3% reported high-level burnout for emotional exhaustion, 20% had high-level burnout for depersonalisation and 10% had high-level burnout for personal achievement (Table 2).

**TABLE 2:** Descriptive statistics of burnout components.

Subscale	Mean	s.d.	High-level burnout		Moderate burnout		Low-level burnout	
			*n*	%	*n*	%	*n*	%
Emotional exhaustion	15.4	± 10.8	5	8.3	15	25.0	40	66.7
Depersonalisation	7.3	± 5.5	12	20.0	17	28.3	31	51.7
Personal achievement	40.0	± 7.8	6	10.0	15	25.0	39	65.0

s.d., standard deviation.

### Burnout and socio-demographic factors

The socio-demographic factors considered were participants’ gender, age, areas of specialty and number of years of practice. There was no statistically significant association between these variables and the various domains of burnout measured (Table 3 and Table 4).

**TABLE 3a:** Analysis of the relationship between socio-demographic data (age and sex) and the levels of burnout: Emotional exhaustion.

Variable	High-level burnout		Moderate burnout		Low-level burnout		Total		*χ*^2^/*b*_px_	*p*
	*n*	%	*n*	%	*n*	%	*n*	%		
**Age (years)**									**112.5**	**0.469**
25–34	1	7.7	4	31.1	8	61.2	13	100.0		
35–44	3	11.5	7	27.0	16	61.5	26	100.0		
45–54	1	7.1	4	28.6	9	64.3	14	100.0		
55–64	0	0.0	0	0.0	5	100.0	5	100.0		
> 64	0	0.0	0	0.0	2	100.0	2	100.0		

**Total**	**5**	**8.3**	**15**	**25.0**	**40**	**66.7**	**60**	**100.0**		

χ^2^, chi-square; *b*_px_, coefficient of regression.

**TABLE 3b:** Analysis of the relationship between socio-demographic data (age and sex) and the levels of burnout: Depersonalisation.

Variable	High-level burnout		Moderate burnout		Low-level burnout		Total		*χ*^2^/*b*_px_	*p*
	*n*	%	*n*	%	*n*	%	*n*	%		
**Age (years)**									**58.2**	**0.680**
25–34	0	0.0	6	46.2	7	53.8	13	100.0		
35–44	6	23.1	7	26.9	13	50.0	26	100.0		
45–54	6	42.9	2	14.2	6	42.9	14	100.0		
55–64	0	0.0	2	40.0	3	60.0	5	100.0		
> 64	0	0.0	0	0.0	2	100.0	2	100.0		

**Total**	**12**	**20.0**	**17**	**28.3**	**31**	**51.7**	**60**	**100.0**		

χ^2^, chi-square; *b*_px_, coefficient of regression.

**TABLE 3c:** Analysis of the relationship between socio-demographic data (age and sex) and the levels of burnout: Personal achievement.

Variable	High-level burnout		Moderate burnout		Low-level burnout		Total		*χ*^2^/*b*_px_	*p*
	*n*	%	*n*	%	*n*	%	*n*	%		
**Age (years)**									**75.3**	**0.501**
25–34	1	7.7	4	30.8	8	61.5	13	100.0		
35–44	2	7.7	4	15.7	20	76.9	26	100.0		
45–54	2	14.3	7	50.0	5	35.7	14	100.0		
55–64	0	0.0	0	0.0	5	100.0	5	100.0		
> 64	1	50.0	0	0.0	1	50.0	2	100.0		

**Total**	**6**	**10.0**	**15**	**25.0**	**39**	**65.0**	**60**	**100.0**		

χ^2^, chi-square; *b*_px_, coefficient of regression.

**TABLE 3d:** Analysis of the relationship between socio-demographic data (age and sex) and the levels of burnout: Depersonalisation.

Variable	High-level burnout		Moderate burnout		Low-level burnout		Total		*χ*^2^/*b*_px_	*p*
	*n*	%	*n*	%	*n*	%	*n*	%		
**Sex**									**30.2**	**0.354**
Male	2	6.6	8	26.7	20	66.7	30	100.0		
Female	3	10.0	7	23.3	20	66.7	30	100.0		

**Total**	**5**	**8.3**	**15**	**25.0**	**40**	**66.7**	**60**	**100.0**		

χ^2^, chi-square; *b*_px_, coefficient of regression.

**TABLE 3e:** Analysis of the relationship between socio-demographic data (age and sex) and the levels of burnout: Depersonalisation.

Variable	High-level burnout		Moderate burnout		Low-level burnout		Total		*χ*^2^/*b*_px_	*p*
	*n*	%	*n*	%	*n*	%	*n*	%		
**Sex**									**10.1**	**0.859**
Male	6	20.0	10	33.3	14	46.7	30	100.0		
Female	6	20.0	7	23.3	17	56.7	30	100.0		

**Total**	**12**	**20.0**	**17**	**28.3**	**31**	**51.7**	**60**	**100.0**		

χ^2^, chi-square; *b*_px_, coefficient of regression.

**TABLE 3f:** Analysis of the relationship between socio-demographic data (age and sex) and the levels of burnout: Personal achievement.

Variable	High-level burnout		Moderate burnout		Low-level burnout		Total		*χ*^2^/*b*_px_	*p*
	*n*	%	*n*	%	*n*	%	*n*	%		
**Sex**									**17.3**	**0.570**
Male	2	6.7	9	30.0	19	63.3	30	100.0		
Female	4	13.3	6	20.0	20	66.7	30	100.0		

**Total**	**6**	**10.0**	**15**	**25.0**	**39**	**65.0**	**60**	**100.0**		

χ^2^, chi-square; *b*_px_, coefficient of regression.

**TABLE 4a:** Analysis of the relationship between socio-demographic data (years of practice and specialty) and the levels of burnout: Emotional exhaustion.

Variable	High-level burnout		Moderate burnout		Low-level burnout		Total		*χ*^2^/*b*_px_	*p*
	*n*	%	*n*	%	*n*	%	*n*	%		
**Number of years of practice**									−**0.11**	**0.302**
1–9	1	5.9	5	29.4	11	64.7	17	100.0		
10–19	3	9.7	9	29.0	19	61.3	31	100.0		
20–29	1	14.3	1	14.3	5	71.4	7	100.0		
30–39	0	0.0	0	0.0	3	100.0	3	100.0		
40–49	0	0.0	0	0.0	2	100.0	2	100.0		

**Total**	**5**	**8.3**	**15**	**25.0**	**40**	**66.7**	**60**	**100.0**		

*χ*^2^, chi-square; *b*_px_, coefficient of regression.

**TABLE 4b:** Analysis of the relationship between socio-demographic data (years of practice and specialty) and the levels of burnout: Emotional exhaustion.

Variable	High-level burnout		Moderate burnout		Low-level burnout		Total		*χ*^2^/*b*_px_	*p*
	*n*	%	*n*	%	*n*	%	*n*	%		
**Number of years of practice**									−**0.05**	**0.820**
1–9	1	5.9	7	41.2	9	52.9	17	100.0		
10–19	9	29.0	8	25.8	14	45.2	31	100.0		
20–29	2	28.5	0	0.0	5	71.5	7	100.0		
30–39	0	0.0	2	66.7	1	33.3	3	100.0		
40–49	0	0.0	0	0.0	2	100.0	2	100.0		

**Total**	**12**	**20.0**	**17**	**28.3**	**31**	**51.7**	**60**	**100.0**		

*χ*^2^, chi-square; *b*_px_, coefficient of regression.

**TABLE 4c:** Analysis of the relationship between socio-demographic data (years of practice and specialty) and the levels of burnout: Personal achievement.

Variable	High-level burnout		Moderate burnout		Low-level burnout		Total		*χ*^2^/*b*_px_	*p*
	*n*	%	*n*	%	*n*	%	*n*	%		
**Number of years of practice**									−**0.12**	**0.420**
1–9	1	5.9	5	29.4	11	64.7	17	100.0		
10–19	3	9.7	9	29.0	19	61.3	31	100.0		
20–29	1	14.3	1	14.3	5	71.4	7	100.0		
30–39	0	0.0	0	0.0	3	100.0	3	100.0		
40–49	1	50.0	0	0.0	1	50.0	2	100.0		

**Total**	**6**	**10.0**	**15**	**25.0**	**39**	**65.0**	**60**	**100.0**		

*χ*^2^, chi-square; *b*_px_, coefficient of regression.

**TABLE 4d:** Analysis of the relationship between socio-demographic data (years of practice and specialty) and the levels of burnout: Emotional exhaustion.

Variable	High-level burnout		Moderate burnout		Low-level burnout		Total		*χ*^2^/*b*_px_	*p*
	*n*	%	*n*	%	*n*	%	*n*	%		
**Specialty**									**195.7**	**0.071**
Community health	0	0.0	0	0.0	3	100.0	3	100.0		
Family medicine	0	0.0	3	37.5	5	62.5	8	100.0		
Internal medicine	3	10.3	9	31.1	17	58.6	29	100.0		
Laboratory medicine	0	0.0	0	0.0	2	100.0	2	100.0		
Paediatrics	2	12.5	3	18.7	11	68.8	16	100.0		
Psychiatry	0	0.0	0	0.0	2	100.0	2	100.0		

**Total**	**5**	**8.3**	**15**	**25.0**	**40**	**66.7**	**60**	**100.0**		

*χ*^2^, chi-square; *b*_px_, coefficient of regression.

**TABLE 4e:** Analysis of the relationship between socio-demographic data (years of practice and specialty) and the levels of burnout: Personal achievement.

Variable	High-level burnout		Moderate burnout		Low-level burnout		Total		*χ*^2^/*b*_px_	*p*
	*n*	%	*n*	%	*n*	%	*n*	%		
**Specialty**									**93.47**	**0.920**
Community health	1	33.3	0	0.0	2	66.7	3	100.0		
Family medicine	1	12.5	2	25.0	5	62.5	8	100.0		
Internal medicine	2	6.9	7	24.1	20	69.0	29	100.0		
Laboratory medicine	0	0.0	0	0.0	2	100.0	2	100.0		
Paediatrics	2	12.5	6	37.5	8	50.0	16	100.0		
Psychiatry	0	0.0	0	0.0	2	100.0	2	100.0		

**Total**	**6**	**10.0**	**15**	**25.0**	**39**	**65.0**	**60**	**100.0**		

*χ*^2^, chi-square; *b*_px_, coefficient of regression.

**TABLE 4f:** Analysis of the relationship between socio-demographic data (years of practice and specialty) and the levels of burnout: Depersonalisation.

Variable	High-level burnout		Moderate burnout		Low-level burnout		Total		*χ*^2^/*b*_px_	*p*
	*n*	%	*n*	%	*n*	%	*n*	%		
**Specialty**									**85.03**	**0.781**
Community health	1	33.3	1	33.3	1	33.3	3	100.0		
Family medicine	1	12.3	3	37.3	4	50.0	8	100.0		
Internal medicine	6	20.7	8	27.6	15	51.7	29	100.0		
Laboratory medicine	0	0.0	1	50.0	1	50.0	2	100.0		
Paediatrics	4	25.0	4	25.0	8	50.0	16	100.0		
Psychiatry	0	0.0	0	0.0	2	100.0	2	100.0		

**Total**	**12**	**20.0**	**17**	**28.3**	**31**	**51.7**	**60**	**100.0**		

*χ*^2^, chi-square; *b*_px_, coefficient of regression.

Amongst the specialties listed, high-level burnout was highest amongst respondents in internal medicine and paediatrics, across all three domains of burnout. These findings were, however, not statistically significant (Table 4).

The greatest proportion of physicians who recorded high-level burnout for all three components (emotional exhaustion, depersonalisation and personal achievements) was those whose years of practice were between 10 and 19 years. The relationship between years of practice and burnout was not statistically significant (Table 4).

## Discussion

The aim of this preliminary study was to determine the prevalence of burnout amongst physicians in Ghana who attended the AGSM, Ghana Chapter, of the WACP. The second objective was to elicit possible associated factors between burnout and age, sex, medical specialty and years of practice. The prevalence of the three components of burnout was 10.8% for emotional exhaustion, 5.5% for depersonalisation and 7.8% for personal achievement. These figures were lower than those obtained, respectively, in Saudi Arabia and Nigeria; 70% and 25.4% for emotional exhaustion; 26% and 9.9% for depersonalisation; and 12% and 31.6% had a low level of personal accomplishment.^2,14^ In an earlier study on Career Satisfaction and Burnout conducted in Ghana, the level of burnout was also found to be low and emotional exhaustion was found to be moderate.^15^ We surmise that the lower prevalence of burnout seems to be a reflection of the Ghanaian physicians’ attitude to work where the satisfaction of the work itself often overrides the challenges they face daily. This situation has been documented with other cadres of health workers in other sub-Saharan countries like Tanzania.^16^ Then again, the vast differences in burnout prevalence rates in these countries may be attributed to the different study designs and sample sizes, different burnout assessment tools and socio-demographic dynamics pertaining to health systems in different countries.^17^

From our study, socio-demographic characteristics such as physician’s age and sex had no statistically significant relationship with their assessment of burnout. However, a previous study explored associations between burnout and physician age, sex and age of children and postulated that looking after children younger than 21 years increased burnout risk by 54%.^18^ A Norwegian study reported that women were found to have higher exhaustion rates which were linked to work–home conflicts.^19^

Furthermore, our study could not establish any association between burnout and years of practice or clinical specialty. Previous data available showed that primary care physicians and emergency physicians have higher rates of burnout as compared with physicians in other specialties.^20^ Reasons proffered for this are the large numbers of clients seen, undifferentiated conditions which require longer consultation periods and the mix of chronic and emergency cases seen daily.^20^

It was quite striking that physicians who had practised between 10 and 19 years had higher levels of burnout for all three dimensions of the MBI, whereas other age groups recorded lower levels (albeit not statistically significant). Although our study did not explore any factors peculiar to this age group, our findings are in contrast to a global study that found that the risk of burnout in physicians younger than 55 years was more than double the risk in those older than 55 years.^21^ The stress of starting a family and the quest for financial stability that compel young physicians to take up multiple job opportunities are thought to heighten the risk of burnout.^14^ However, the situation of higher burnout amongst the older physicians in Ghana could be explained by the feeling of inadequate preparation for impending retirement, financial and other demands of extended family, societal expectations, career or academic expectations, which are all heavy strains on the Ghanaian physician. Retirement with a pension in Ghana was found to be protective from major depressive illness only in males aged 60 years and above in a recent cross-sectional study.^22^

These findings underscore the need for well-structured career guidance and counselling for secondary and tertiary-level students as well as young doctors. It is imperative that the choice of a medical profession or clinical specialty should be guided by a person’s appreciation of unique characteristics, strengths, resilience and coping skills. Policymakers and health system administrators should also institute measures to prevent burnout at the workplace and support staff members who suffer from this. This would ensure efficient and increased work output, staff and patient satisfaction.

### Limitations and strengths

Our ability to make generalisations from this study was limited by the relatively small sample size and the fact that it involved only participants attending a conference. The sample size also contributed to the statistically insignificant *p*-values for the measured variables. However, because it was a pilot study and the participants were physicians who are at the cutting edge of their careers, the findings provide a useful reference for future studies. We recommend further research involving a larger population of physicians and the exploration of various factors related to medical specialists and consultants.

## Conclusion

Our study has demonstrated that burnout within the medical fraternity is prevalent and remains relevant, although it is a relatively unexplored subject in much of sub-Saharan Africa. Despite the fact that there were no statistically significant associations between burnout and sex, age, medical specialty and years of practice, continuous efforts should be made to identify burnout-related factors at the workplace and build capacity of physicians to address them. Our findings are useful for future studies that should recruit a larger study population across the medical and surgical specialties. This would permit a more rigorous testing of the variables as well as other key research questions to enhance the current body of knowledge and influence policy shift where required.
